# Establishment and validation of a prognostic nomogram for patients with early-onset stage I–II colon cancer

**DOI:** 10.1186/s12957-023-02988-w

**Published:** 2023-03-24

**Authors:** Dongdong Li

**Affiliations:** Department of General Surgery, Xinhua Hospital, Shanghai Jiao Tong University School of Medicine, Shanghai, China

**Keywords:** Colorectal cancer, Early-Onset, Prognostic factor, Nomogram

## Abstract

**Background:**

The aims of this study were to establish and validate a nomogram model for predicting the survival of patients with early-onset stage I–II colon cancer (CC).

**Methods:**

Data of eligible patients enrolled from 2012 to 2015 were extracted from the Surveillance, Epidemiology, and End Results (SEER) database. Patients were randomly allocated to training and validation groups in a 7:3 ratio. Significant prognostic factors were identified by univariate and multivariate analysis and a nomogram model constructed. The predictive performance of the nomogram was evaluated by the concordance index (C-index), calibration plots, and decision curve analysis.

**Results:**

Our study cohort comprised 3528 early-onset CC patients with stage I–II disease, 2469 of whom were allocated to the training cohort and 1059 to the validation cohort. Race, age, marital status, tumor grade, tumor size, tumor stage (T stage), and chemotherapy were considered the significant predictor by univariate analysis. Race, marital status, and T stage were found to be independent prognostic factors by multivariate analysis. The C-indexes of the nomogram were 0.724 and 0.692 in the training and validation cohorts, respectively. Likewise, the calibration plots showed good agreement regarding the probability of 3- and 5-year observed and nomogram-predicted overall survival in the training group. Decision curve analysis showed that the nomogram model was clinically practical and effective. Moreover, applying the nomogram enabled dividing of the patients into two cohorts with different risk scores. The low-risk group thus created had a better survival than the high-risk group.

**Conclusions:**

We developed and validated a meaningful prognostic nomogram model for patients with early-onset stage I–II CC that clinicians can use to make better decisions for individual patients.

## Introduction

Colorectal cancer (CRC) has become the third most common cancer and the second leading cause of cancer-related mortality worldwide. Colon cancer (CC) accounts for a large proportion of CRC [1, 2]. The increased implementation of screening has resulted in an increase in the number of newly diagnosed patients with early-stage CC [3]. Although this is generally considered to provide an opportunity for curative-intent treatment, the prognosis of some patients remains poor. Of particular interest, the incidence of early-onset CRC (defined as CRC occurring under the age of 50 years) has been increasing in many countries [4–6]. This has resulted in a heavy cancer burden in younger adults. Hence, predicting the prognosis of these patients warrants investigation.

The Tumour, Node, Metastasis (TNM) staging system is regarded as providing a helpful prognostic index for CC patients, being useful for predicting their clinical outcomes from the point of view of tumor biology and anatomy [7]. Even so, it may not be the optimal prognostic indicator. The roles of other risk factors, such as race [8], age [9], sex [10], tumor site [11], tumor size, and chemotherapy administered [12], that affect the prognosis of CC patients should not be ignored. In other words, it is necessary to use a combination of possible influencing factors to predict the survival of cancer patients more accurately.

The Surveillance, Epidemiology, and End Results (SEER) database contains much information about cancer-related risk factors and patients’ survival. It is crucial to synthesize this information wisely. Nomograms, being a statistical prognostic model, can integrate diverse biologic and clinical variables to generate an individual’s probability of experiencing a clinical event, thus facilitating achieving the goal of providing personalized medicine [13]. To the best of our knowledge, no researchers have used data drawn from the SEER database to construct a nomogram model for predicting the prognosis of patients with early-onset stage I–II CC.

In this study, we aimed to establish a novel model that includes multiple variables and thus more accurately predicts the survival of patients with early-onset, early-stage CC. This nomogram should enable clinicians to make better treatment decisions for such individuals.

## Methods

The data were obtained from the SEER Program, which is dedicated to collecting and providing cancer statistics with the aim of reducing the cancer burden in the USA. We used data collected from 2012 to 2015. These data included baseline patient and tumor characteristics and survival information. The inclusion criteria for this study were (a) age under 50 years; (b) surgery performed; (c) postoperative pathological diagnosis of stage I–II CC without distance metastasis; and (d) ≥ 12 regional nodes examined. The exclusion criteria were (a) no prior tumor; (b) unknown histological grade; (c) unknown marital status; (d) unknown race; (e) death from other tumors and unknown cause of death; and (f) survival time recorded as zero. Ultimately, our study cohort comprised 3528 patients with early-onset stage I–II CC.

These following variables were extracted and included in the analysis: baseline patient characteristics (race, sex, age at diagnosis, survival [months], marital, and vital status), tumor features (tumor site, pathological grade, tumor size, TNM stage, and T stage), and treatment strategy (chemotherapy). Staging was in accordance with the seventh edition of the American Joint Committee on Cancer (AJCC) TNM classification. Race was classified as white, black, or other. Sex was stratified as male or female. Two age groups were created: ≤ 35 and > 35 years. Pathological grades I–IV were categorized as well differentiated, moderately differentiated, poorly differentiated, and undifferentiated. Additional study variables comprised tumor site (left or right side), chemotherapy (no or yes), marital status (married or unmarried), tumor size (≤ 5 cm or > 5 cm) and T stage (T1, T2, T3, T4). Overall survival (OS) time was defined as the time from diagnosis to death from any cause.

All eligible patients were randomly allocated to a training (*n* = 2469) or validation group (*n* = 1059) in a 7:3 ratio. The training group was used to construct the nomogram and the validation group for validation. Univariate and multivariate regression analysis were applied to identify the factors that significantly affected the patients’ OS (*p* < 0.05). The nomogram model was created using R software (version 3.6.1) and the identified significant variables. The performance and predictive accuracy of the nomogram were evaluated by the concordance index (C-index). The C-index ranges from 0.5 to 1.0, where the larger the value, the more accurately the nomogram model predicts outcomes. Calibration plots were drawn at 3 and 5 years to compare the predicted with the actual OS. Decision curve analyses (DCA) were performed to evaluate the clinical practicability of the nomogram. The median score calculated from the nomogram among the training cohort was set as the cutoff value. Thus, all eligible patients were classified into two groups (low versus high score). Kaplan–Meier curves and the log-rank test were used to compare the OS between groups. We used IBM SPSS Statistics, Version 25.0 (SPSS) to perform all univariate and multivariate regression analyses and constructed the graphs using R software and related packages. *P* values less than 0.05 were considered to denote statistical significance.

## Results

### Patient’s baseline characteristics

The patients’ baseline characteristics are summarized in Table 1. A total of 3528 patients with early-onset stage I–II CC were included in our study: 2469 patients in the training cohort and 1059 in the validation cohort. There were no significant differences in assessed characteristics between the two groups (all *p* > 0.05). In the entire cohort, 52% of patients (*n* = 1834) were male, 89.5% (*n* = 3159) were aged > 35 years, 73.5% (*n* = 1834) were white, and 55.8% (*n* = 1969) were married. More than half the patients had tumors bigger than 5 cm and located on the left side. The cancers were pathological grades I/II in 3090 (87.6%) and stage T3/T4 in 2046 (58.0%) patients, and 763 patients (21.6%) had received chemotherapy.

**Table 1:** Baseline characteristics of patients in the training and validation cohorts

	All cohort	Training cohort	Validation cohort	
	*n* = 3528	*n* = 2469	*n* = 1059	
Characteristic	*N* (%)	*N* (%)	*N* (%)	*P* value
Sex				0.941
Female	1694(48.0)	1184(48.0)	510(48.2)	
Male	1834(52.0)	1285(52.0)	549(51.8)	
Age, years				0.880
> 35	3159(89.5)	2209(89.5)	950(89.7)	
≤ 35	369(10.5)	260(10.5)	109(10.3)	
Race				0.798
Black	553(15.7)	392(15.9)	161(15.2)	
Other	380(10.8)	269(10.9)	111(10.5)	
White	2595(73.5)	1808(73.2)	787(74.3)	
Marital status				0.097
Married	1969(55.8)	1355(54.9)	614(58.0)	
Unmarried/NOS	1559(44.2)	1114(45.1)	445(42.0)	
Tumor site				0.975
Left	1912(54.2)	1339(54.2)	573(54.1)	
Right	1616(45.8)	1130(45.8)	486(45.9)	
Grade				0.974
Grade I	377(10.7)	261(10.6)	116(11.0)	
Grade II	2713(76.9)	1899(76.9)	814(76.9)	
Grade III	346(9.8)	245(9.9)	101(9.5)	
Grade IV	92(2.6)	64(2.6)	28(2.6)	
Tumor size (cm)				0.149
> 5	1579(44.8)	1085(43.9)	494(46.6)	
≤ 5	1949(55.2)	1384(56.1)	565(53.4)	
T				0.454
T1	897(25.4)	638(25.8)	259(24.4)	
T2	585(16.6)	406(16.4)	179(16.9)	
T3	1698(48.1)	1172(47.5)	526(49.7)	
T4	348(9.9)	253(10.2)	95(9.0)	
TNM				0.636
I	1482(42.0)	1044(42.3)	438(41.4)	
II	2046(58.0)	1425(57.7)	621(58.6)	
Chemotherapy				0.962
Yes	763(21.6)	535(21.7)	228(21.5)	
No/unknown	2765(78.4)	1934(78.3)	831(78.5)	
Survival status				0.348
Alive	3377(95.7)	2369(95.9)	1008(95.2)	
Dead	151(4.3)	100(4.1)	51(4.8)	

### Identification of significant prognostic factors by univariate and multivariate analysis

The results of univariate and multivariate analysis in the training cohort are shown in Table 2. Univariate analysis identified race, age, marital status, tumor grade, tumor size, T stage, and chemotherapy as significant predictors of OS (all *p* < 0.05). Multivariate analysis of these factors identified race, marital status, and T stage as independent prognostic factors. Accordingly, these variables were used to construct the nomogram model.

**Table 2:** Results of univariate and multivariate analysis of potential prognostic factors in the training cohort

	Univariate analysis		Multivariate analysis	
Characteristic	HR (95%CI)	*P* value	HR (95%CI)	*P* value
Race				
White	Reference		Reference	
Black	2.322(1.510–3.571)	< 0.001	1.992(1.286–3.085)	0.002
Other	0.623(0.270–1.440)	0.268	0.607(0.262–1.405)	0.244
Sex				
Female	Reference			
Male	1.145(0.772–1.700)	0.501		
Age				
≤ 35	Reference		Reference	
> 35	0.560(0.333–0.944)	0.030	0.696(0.407–1.192)	0.187
Marital status				
Married	Reference		Reference	
Unmarried/NOS	2.340(1.551–3.529)	< 0.001	1.888(1.239–2.879)	0.003
Tumor site				
Right	Reference			
Left	1.056(0.712–1.568)	0.786		
Grade				
Grade I	Reference		Reference	
Grade II	1.186(0.571–2.463)	0.648	0.969(0.463–2.029)	0.934
Grade III	1.476(0.603–3.610)	0.394	0.993(0.398–2.477)	0.988
Grade IV	4.891(1.886–12.679)	0.001	2.509(0.938–6.714)	0.067
Tumor size (cm)				
≤ 5	Reference		Reference	
> 5	1.544(1.043–2.287)	0.030	1.081(0.713–1.639)	0.714
T				
T1	Reference		Reference	
T2	2.107(0.957–4.642)	0.064	2.119(0.957–4.688)	0.064
T3	2.407(1.247–4.647)	0.009	2.224(1.119–4.421)	0.023
T4	7.775(3.883–15.571)	< 0.001	6.358(2.849–14.190)	< 0.001
Chemotherapy				
Yes	Reference		Reference	
No/unknown	0.485(0.323–0.730)	0.001	1.055(0.640–1.738)	0.834

### Construction and validation of the nomogram

**Fig. 1:**
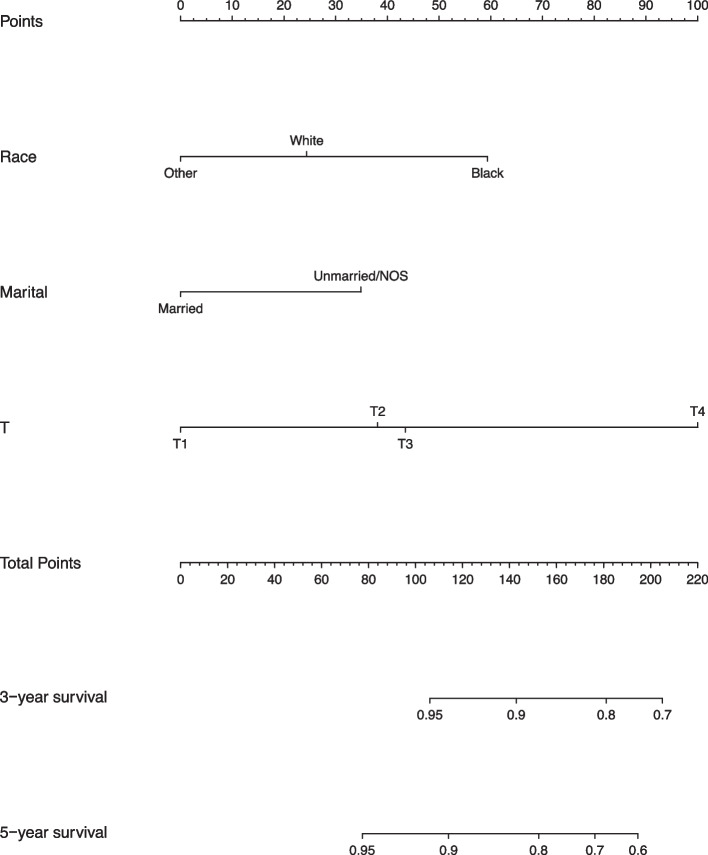
Nomogram for predicting overall survival of patients with early-onset stage I–II colon cancer

**Fig. 2:**
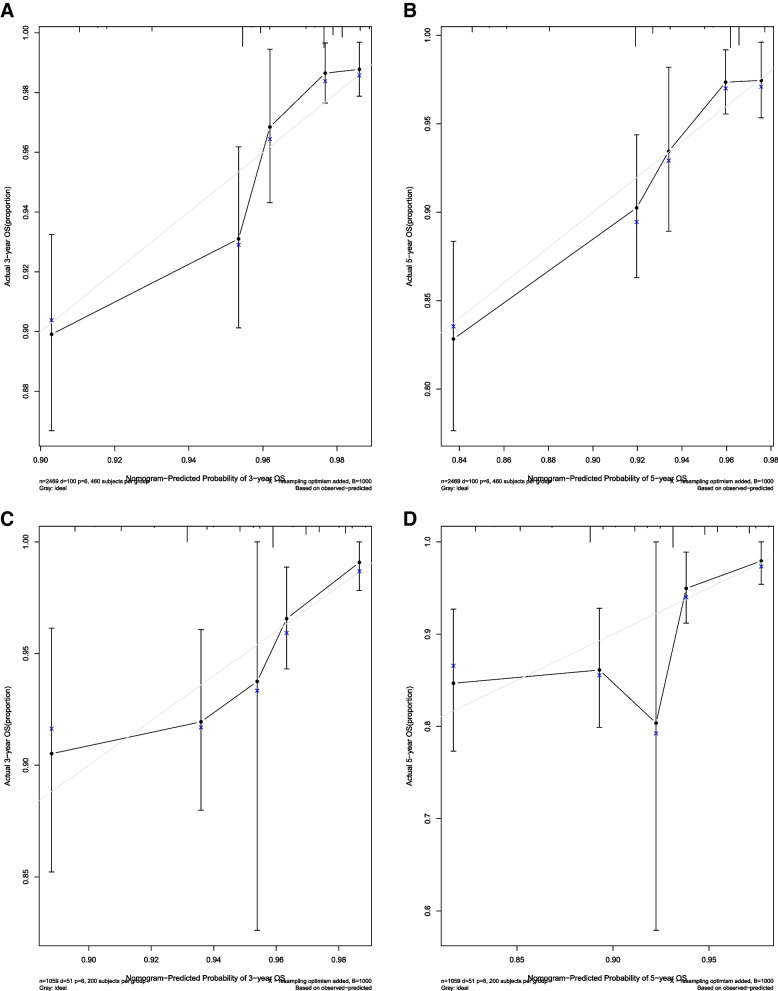
Calibration curves predicting 3- and 5-year OS in the training and validation group. **A** Calibration curve predicting 3-year OS in the training group. **B** Calibration curve predicting 5-year OS in the training group. **C** Calibration curve predicting 3-year OS in the validation group. **D** Calibration curve predicting 3-year OS in the validation group.OS, overall survival

**Fig. 3:**
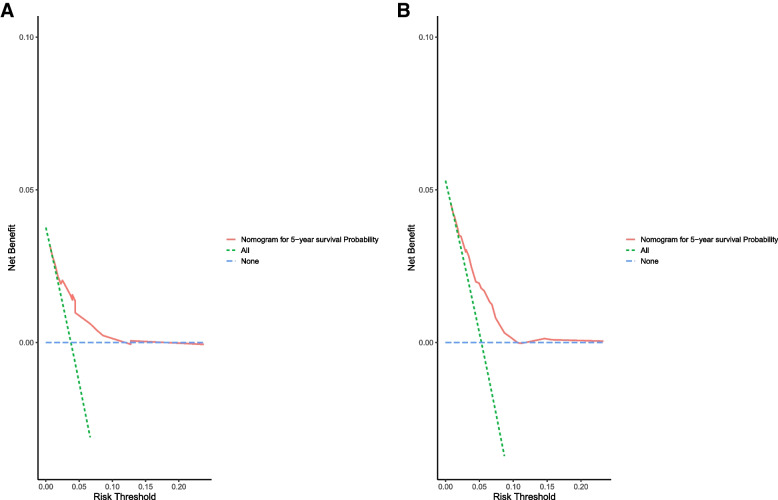
Results of decision curve analysis of OS-associated nomogram in training and validation groups. **A** Results of decision curve analysis curve of 5-year OS in the training group. **B** Results of decision curve analysis curve of 5-year OS in the validation cohort. OS, overall survival

In accordance with the results of multivariate analysis, race, marital status, and T stage were used to build a nomogram for predicting the 3- and 5-year OS (Fig. 1). Each predictor was assigned a score, ranging from 0 to 100. The nomogram showed that T stage was the dominant contributor to the OS, followed by race and marital status. Total scores for specific patients were calculated by adding the scores for each variable. The chances of 3- and 5-year OS were obtained by drawing a vertical line through the location of the total score on the horizontal axis. The C-index of the nomogram for the training cohort was 0.724. The calibration curves showed good consistency in the probability of 3- and 5-year OS between the observed and nomogram-predicted outcomes in the training cohort (Fig. 2A, B). Further, the DCA curves for the training cohort showed that the nomogram model was practical and effective (Fig. 3A). We then used the same procedure to verify the nomogram model in the validation cohort. The C-index in the validation cohort was 0.692. Likewise, the calibration curves (Fig. 2C, D) and the DCA curves (Fig. 3B) in the validation cohort showed that the nomogram was robust and applicable.

### Comparison of survival differences between groups with different scores based on the nomogram

**Fig. 4:**
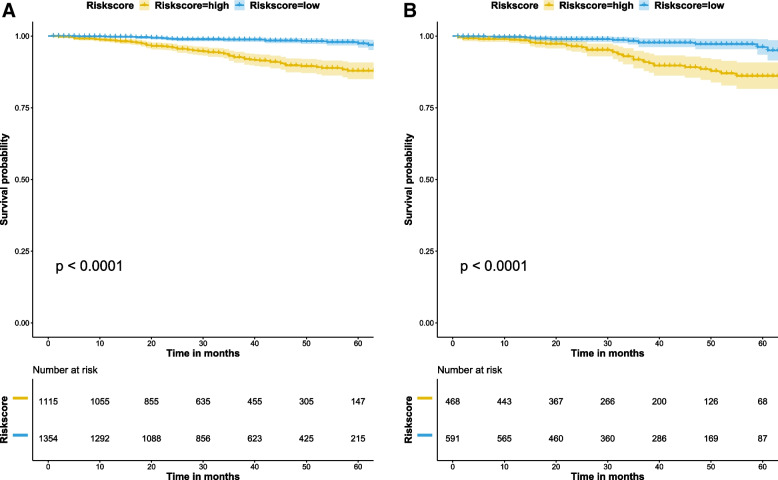
Survival curves of OS for risk classification based on the nomogram risk score. **A** In the training group. **B** In the validation group. OS, overall survival

After determining that the nomogram had good predictive value, we wanted to distinguish the patients’ OS according to their scores. Accordingly, we stratified the patients into two groups based on the cutoff value, that is, the median of the total scores in the training cohort. In the training cohort, patients with low-risk scores (score < 73.15) had a better OS than those with high-risk scores (score ≥ 73.15) (*P* < 0.001) (Fig. 4A). Likewise, we determined that the survival curves differed significantly in the validation set (*p* < 0.001) (Fig. 4B).

## Discussion

As is well known, the incidence of early-onset CRC is on the rise. The reasons for this trend remain unclear. Moreover, some patients with early-stage disease do not achieve a satisfactory outcome despite undergoing surgery. We therefore selected eligible patients from the SEER database with the aim of developing and validating a prognostic nomogram model for patients with early-onset stage I–II CC and established that this nomogram has good prognostic value.

In our study, univariate and multivariate analysis identified T stage, race, and marital status as the most significant predictors of OS. It is well established that, in patients with early-stage solid tumors without lymph node or distant metastases, the T stage of the TNM staging system makes a major contribution to determining prognosis [14, 15]. Previous research has shown that T stage is an independent predictor among many variables in patients with CRC. That is, the higher the T stage, the lower the survival rate [8, 16]. Li et al. found that the T stage has greater weight than the N stage in the TNM staging system for CRC; that is, the T stage affects survival from CRC more significantly than does the N stage [17]. Consistent with this, according to our nomogram, of the studied variables, T stage had the greatest impact on OS. In other words, the higher the T stage, the worse the OS.

In addition, our nomogram identified that race is significantly associated with survival, patients in the “other” category having a higher survival rate than those categorized as white or black. Previous research on advanced CC has had similar results [18]. However, a SEER-based study on early hepatocellular carcinoma found that those categorized as white have better survival rates than those categorized as black or other [19]. We speculate that this discrepancy may be related to factors such as the type of cancer, genetics and genomic context of different selected patients.

Another significant variable identified by our nomogram was marital status; this is consistent with the findings of other studies that married patients have survival advantages [20, 21]. We also found that married patients have a higher chance of survival than unmarried patients. A stable family may provide better care and psychological support, enhancing quality of life and improving survival.

The prognostic risk of patients with early-onset early-stage CC can be quantified relatively on the basis of these three variables. To our knowledge, few studies have focused on and explored this question. However, variables not included in the model should not be ignored. They may also affect prognosis under certain conditions that are yet to be determined [12, 22].

Our study had some limitations. First, it was retrospective; the data came from a public database and had not been validated in the real world. Second, some potentially relevant details, such as molecular markers, molecular pathological features of tumor, surgical procedures, inflammatory and tumor indicators, and specifics of postoperative treatment, were not available, possibly resulting in bias. Finally, the nomogram and risk classification system should be further verified in another institution.

## Conclusions

In this paper, we identified predictors of prognosis and used them to develop a useful a nomogram model for predicting the OS of patients with early-onset, stage I–II CC. This nomogram has the potential to help clinicians make treatment decisions. However, external validation is still required.

## Data Availability

The data sets analyzed in this study are available on the public databases.

## Abbreviations

AJCC: American Joint Committee on Cancer
CC: Colon cancer
CRC: Colorectal cancer
C-index: Concordance index
DCA: Decision curve analysis
OS: Overall survival
SEER: Surveillance, Epidemiology, and End Results
TNM: Tumour, node, metastasis
T stage: Tumor stage

## References

[1] Siegel RL, Miller KD, Fuchs HE, Jemal A (2022). Cancer statistics, 2022. CA Cancer J Clin.

[2] Xia C, Dong X, Li H (2022). Cancer statistics in China and United States, 2022: profiles, trends, and determinants. Chin Med J (Engl).

[3] Buccafusca G, Proserpio I, Tralongo AC, Rametta Giuliano S, Tralongo P (2019). Early colorectal cancer: diagnosis, treatment and survivorship care. Crit Rev Oncol Hematol.

[4] Mauri G, Sartore-Bianchi A, Russo AG, Marsoni S, Bardelli A, Siena S (2019). Early-onset colorectal cancer in young individuals. Mol Oncol.

[5] Sinicrope FA (2022). Increasing Incidence of Early-Onset Colorectal Cancer. N Engl J Med.

[6] Patel SG, Karlitz JJ, Yen T, Lieu CH, Boland CR (2022). The rising tide of early-onset colorectal cancer: a comprehensive review of epidemiology, clinical features, biology, risk factors, prevention, and early detection. Lancet Gastroenterol Hepatol.

[7] Amin MB, Greene FL, Edge SB (2017). The Eighth Edition AJCC Cancer Staging Manual: Continuing to build a bridge from a population-based to a more "personalized" approach to cancer staging. CA Cancer J Clin.

[8] Liu Z, Xu Y, Xu G (2021). Nomogram for predicting overall survival in colorectal cancer with distant metastasis. BMC Gastroenterol.

[9] Boakye D, Walter V, Jansen L (2020). Magnitude of the Age-Advancement Effect of Comorbidities in Colorectal Cancer Prognosis. J Natl Compr Canc Netw.

[10] Kim SE, Paik HY, Yoon H, Lee JE, Kim N, Sung MK (2015). Sex- and gender-specific disparities in colorectal cancer risk. World J Gastroenterol.

[11] Temraz S, Mukherji D, Nassar F, Moukalled N, Shamseddine A (2021). Treatment sequencing of metastatic colorectal cancer based on primary tumor location. Semin Oncol.

[12] Palmieri LJ, Fihri A, Doat S (2019). Tumor-size responses to first-line is a predictor of overall survival in metastatic colorectal cancer. Eur Radiol.

[13] Kong X, Li J, Cai Y (2018). A modified TNM staging system for non-metastatic colorectal cancer based on nomogram analysis of SEER database. BMC Cancer.

[14] Shin JY, Yoon JK, Marwaha G (2018). Progress in the treatment and outcomes for early-stage non-small cell lung cancer. Lung.

[15] Wen C, Tang J, Luo H (2022). Development and validation of a nomogram to predict cancer-specific survival for middle-aged patients with early-stage hepatocellular carcinoma. Front Public Health.

[16] Wu J, Lu L, Chen H (2021). Prognostic nomogram to predict the overall survival of patients with early-onset colorectal cancer: a population-based analysis. Int J Colorectal Dis.

[17] Li J, Guo BC, Sun LR (2014). TNM staging of colorectal cancer should be reconsidered by T stage weighting. World J Gastroenterol.

[18] Li Y, Liu W, Zhou Z (2020). Development and validation of prognostic nomograms for early-onset locally advanced colon cancer. Aging (Albany NY).

[19] Yan B, Su BB, Bai DS (2021). A practical nomogram and risk stratification system predicting the cancer-specific survival for patients with early hepatocellular carcinoma. Cancer Med.

[20] Chen ZH, Yang KB, Zhang YZ (2021). Assessment of modifiable factors for the association of marital status with cancer-specific survival. JAMA Netw Open.

[21] Wang X, Cao W, Zheng C, Hu W, Liu C (2018). Marital status and survival in patients with rectal cancer: An analysis of the Surveillance, Epidemiology and End Results (SEER) database. Cancer Epidemiol.

[22] Loree JM, Pereira AAL, Lam M (2018). Classifying Colorectal Cancer by Tumor Location Rather than Sidedness Highlights a Continuum in Mutation Profiles and Consensus Molecular Subtypes. Clin Cancer Res.

